# Transcriptome Sequencing and Bioinformatics Analysis of Ovarian Tissues from Pomacea canaliculata in Guangdong and Hunan

**DOI:** 10.1155/2022/3917036

**Published:** 2022-04-06

**Authors:** Jing Liu, Jian Li, Zhi Wang, Hua Yang

**Affiliations:** ^1^College of Resources and Environment, Hunan Agriculture University, Changsha, Hunan 410128, China; ^2^College of Bioscience and Biotechnology, Hunan Agriculture University, Changsha, Hunan 410128, China; ^3^College of Life Sciences, Hunan Normal University, Changsha, Hunan 410081, China

## Abstract

In this study, the fecundity of Pomacea canaliculata was studied by collecting egg masses from Guangdong and Hunan using field egg collection and indoor propagation. Through high-throughput RNA sequencing (RNA-seq), we analyzed the ovarian tissue of the snails in Guangdong (G_O) and those in Hunan (H_O) using comparative analysis of transcription. Moreover, we used bioinformatics methods to screen the key pathways and genes that affect the fecundity of snails from the two locations. *Results.* The results showed that the absolute fecundity and weight-relative fecundity of Pomacea canaliculata in Guangdong were significantly higher than those in Hunan. We found 1,546 differential genes through differential gene screening (528 genes upregulated in snails from Guangdong and 1018 in snails from Hunan). The ribosomal signaling pathway and *rpl23a*, *uba52* are critical pathways and essential genes that affect the fecundity of snails. *Conclusions*. The 27 differential genes in the ribosome signaling pathway, collected from H_O, were all downregulated. As a result, ovarian tissue protein synthesis is impaired, which is an important mechanism that affects snails' ability to reproduce.

## 1. Introduction

Invasive species are one of the main threats to biodiversity. Many freshwater snails are likely to damage the function or structure of the ecosystem [1]. However, only one type of snail is listed as one of the world's 100 most invasive species by the International Union for Conservation of Nature [2]. Pomacea canaliculata, also known as apple snails, is native to the Amazon River Basin. In the early 1980s, people introduced it into China and other Asian countries as a high-protein food for commercial purposes. However, they were discarded in large quantities due to their unpalatable taste and poor market sales. Eventually, they settled in the natural environment. The snails seriously endanger aquatic crops, such as rice, and xerophyte crops, such as vegetables near waters, causing considerable losses to the agricultural economy and a severe threat to ecological security.

The level of biological individual fecundity reflects the adaptation characteristics of species or populations to environmental changes, which directly affects the replenishment and proliferation of populations [3]. The vital fecundity of the snails provides favorable conditions for the formation of invasion hazards [4]. A female apple snail can lay 50–800 eggs at a time. On average, each female apple snail can lay 13,764 eggs in its lifetime and reproduce 6,070 young apple snails [5]. The feeding ratio of female snails is higher than that of male snails, so more energy is introduced into the female snail population. When food is limited, female snails use more energy for reproduction. Only if the food supply is reduced above 50% will the fecundity of the snails decrease and be partially compensated for by the increased hatching survival rate [6]. In the long-term restriction of 80% of the food supply, snails can still mature, mate, and lay viable eggs [7]. In addition, the number of female snails in the snail population tends to be more than that of male snails [5], and female snails can lay eggs multiple times after mating once [8]. These reproductive characteristics of Pomacea canaliculata enable them to quickly adapt to the environment they invade where they dominate.

This study investigated Pomacea canaliculata samples from Guangdong and Hunan. Their reproductive characteristics were studied using field survey sampling and laboratory spawning. The differentially expressed genes were enriched and analyzed based on the ovarian transcriptome data, and genes related to reproduction were screened. Research on the fecundity of snails can help clarify the law of population growth and reproductive strategies, which might provide a specific theoretical reference for predicting its spread and provide scientific guidance for the prevention and control of apple snails.

## 2. Materials and Methods

### 2.1. Experimental Materials

The *P*. canaliculata used in this research were collected from the field. The *P.* canaliculata in Guangdong were collected in rice fields in Gaoyao District, Zhaoqing City, Guangdong Province. In contrast, the P. canaliculata in Hunan were collected from ponds in Furong District, Changsha City, Hunan Province. The two snail breeds were separately reared in aquariums at room temperature according to sex (i.e., male and female). The water in the box was tap water aired outdoors for two days and fed with cabbage. The experiment was reviewed and approved by the Ethics Committee of Hunan Agricultural University, and it followed all the principles of care and use of laboratory animals.

### 2.2. Determination of the Reproductive Ability of Snails

#### 2.2.1. Determination of the Reproductive Ability of Snails in the Field

In October 2020, we randomly collected 30 complete egg masses of snails from the collection sites of snails in Guangdong and Hunan. To avoid damaging the collected samples, we took them out together with the attachments of the snail egg masses and brought them back to the laboratory. We then soaked them in a 2% sodium hydroxide solution until the eggs were separated from the attachments, which helped us count the number of eggs.

#### 2.2.2. Determination of the Reproductive Ability of Snails under Laboratory Conditions

Using a plastic bucket (D24 cm∗H60 cm) as the experimental container, we put the snails from Guangdong and Hunan together for single-pair mating. First, we selected snails with normal vitality and no damage to their body surfaces. Then, we absorbed the water on their body surfaces using gauze and measured their mass (W, g). Finally, we placed male and the female pair in the experimental barrel for single-pair mating. Each mating group had three repetitions. The experiment lasted one month. We used cabbage as bait, changed the water every two days, kept the water temperature in the biochemical incubator at 25 ± 1°C, and covered the bucket's mouth with gauze to prevent the snails from escaping. We also observed the living conditions of snails every day and recorded the date and number of spawning eggs. When the surface of the egg mass became hard while the connection between it and the attachment was still wet, we gently peel it off with a small blade. We then soaked it in a 2% sodium hydroxide solution until the egg pieces were entirely dispersed, allowing us to count the number of egg particles.

### 2.3. RNA Extraction and Transcriptome Sequencing

This study took female apple snails for transcriptome sequencing from that had completed spawning. After dissection of the snail, the ovarian tissue was rapidly excised, snap-frozen in liquid nitrogen, and stored at -80°C for RNA extraction. We named snails in Guangdong G_O and snails in Hunan H_O. We generated three biological replicates of snails in each area. The total RNA was extracted with an RNA extraction kit, and an Agilent 2100 bioanalyzer detected the quality and concentration of the total RNA. Novogene Co., Ltd. constructed the sequencing library, and transcriptome paired-end sequencing was performed using the Illumina HiSeq2500 platform.

### 2.4. Screening Differential Genes

We obtained clean reads by removing the sequences containing linkers and low-quality sequences from the original sequence data. The reference genomes were downloaded from the NCBI database and aligned with the Tophat software. The DESeq2 R software was used to screen for differential expression of genes, and differential genes were defined as FDR < 0.05 and FC > 2.

### 2.5. GO Function Annotation of Differential Genes and Enrichment Analysis of the KEGG Pathway

The study also used the Cytoscape plug-in ClueGo + Cluepedia to analyze the differential genes GO (gene ontology) and KEGG (Kyoto encyclopedia of genes and genomes). The analysis parameter setting adopts the software default setting, the network specificity selection is medium, and the result is displayed when *p* < 0.05.

### 2.6. Analysis of the Protein Interaction Network of Differential Genes

The study used the STRING online analysis software for the protein-protein interaction network analysis of differential genes. In order to make the related protein interaction network closer to the real functional state, only the nodes with interaction score greater than 0.9 were retained, and the nodes with failed gene symbol identification were deleted. The protein interaction network was visualized using the Cytoscape software, and the network topology was analyzed using the CytoHubba plug-in to screen out hub genes.

### 2.7. Statistical Analysis

We processed the data using the Microsoft Excel 2016 software, and the results were expressed as Mean ± Standard Deviation. Moreover, we also used the SPSS 26 and a one-way analysis of variance (ANOVA) to analyze the differences between groups (*p* < 0.05). The calculation formula is absolute fecundity = eggs/egg mass; relative weight fecundity = eggs/weight.

## 3. Results

### 3.1. Comparison of the Reproductive Ability of Snails

The experimental results conducted on snails from Guangdong and Hunan are shown in Table 1. The results show significant differences in the absolute fecundity of Pomacea canaliculata in Guangdong and Hunan. In addition, there is no significant difference in the number of egg-laying snails in Guangdong and Hunan. However, there were substantial differences in the number of eggs and relative weight and fecundity (see Table 2).

The same letter indicates that the difference is not significant, and the different letter suggests that the difference is significant (*p* < 0.05).

The exact number and letters in the same column indicate that the difference is insignificant, and different letters indicate a significant difference (*p* < 0.05).

### 3.2. Assessing Sequencing Data Quality

After processing the sequencing data, we obtained 312,760,252 clean reads. Based on the data, the clean bases of each sample were above 7.23 Gb, while the mapped ratio was above 80%. The comparison ratio with the reference genome was between 80.51% and 85.91%, and the unique comparison ratio was higher than 77.91% (see Table 3).

### 3.3. Screening of Differentially Expressed Genes

**Figure 1:**
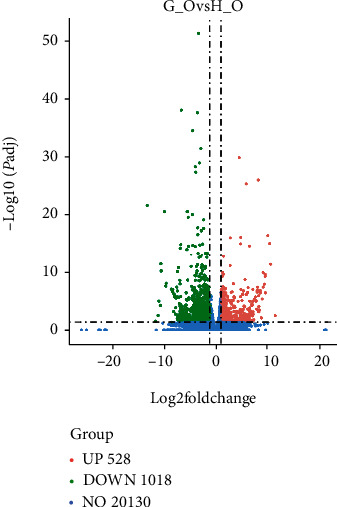
Volcano plot of differentially expressed genes. The red dots in the volcano map indicate upregulated genes, green dots indicate downregulated genes, and blue dots indicate indifferent genes.

Comparing G_O and H_O through differential expression analysis, 1,546 differentially expressed genes were obtained. Compared with H_O, there were 528 genes upregulated and 1018 genes downregulated in G_O. From the volcano map of differentially expressed genes from G_O vs. H_O (Figure 1), we can quickly see that the farther the deviation from 0 is for the *x*-axis, the more significant the considerable difference in expression. For the *y*-axis, the larger the value, the smaller the probability of false positives, and the more reliable the result.

### 3.4. Differential Gene GO Function Enrichment and KEGG Pathway Analysis

**Figure 2:**
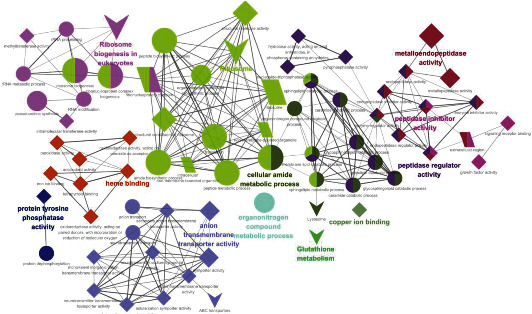
Network of enriched GO terms and KEGG pathways. The circle represents BP, the diamond represents MF, the quadrilateral represents CC, and the arrow represents the pathway. Based on the kappa score level (≥0.1), the term is used as a functional grouping network of connecting nodes. For each group, the size of the nodes indicates their importance, and the largest node size represents the most critical path.

**Figure 3:**
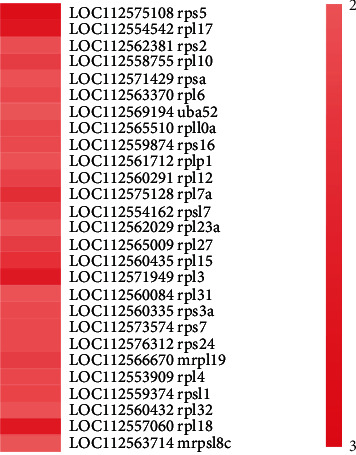
Expression profile of differentially expressed genes annotated with ribosomal signaling pathways. The bar's color represents the fold of upregulation of the corresponding gene. The darker the color, the larger the value, indicating that the fold of the gene is upregulated.

The 66 GO entries in 5 KEGG channels are divided into 12 groups and connected by 144 edges. The most important terms include GO:0005507 copper ion binding (*p* = 0.00764, enrichment number 7), dre00480 Glutathione metabolism (*p* = 0.01249, enrichment number 7), GO:1901564 organonitrogen compound metabolic process (*p* = 0.002356, enrichment number 49), and GO:0004725 protein tyrosine phosphatase activity (*p* = 0.01875, enrichment number 9),

GO:0030414 peptidase inhibitor activity (*p* = 0.00693, enrichment number 13), GO:0020037 heme-binding (*p* = 0.01557, enrichment number 22), dre03008 ribosome biogenesis in eukaryotes (*p* = 2.07 × 10^−7^, enrichment number 17), GO:0004222 metalloendopeptidase activity (*p* = 0.00018, enrichment number 16), GO:0043603 cellular amide metabolic process (*p* = 0.00001, enrichment number 33), GO:0008509 anion transmembrane transporter activity (*p* = 0.00380, enrichment number 10), GO:0061134 peptidase regulator activity (*p* = 0.00693, enrichment number 13), dre03010 Ribosome (*p* = 1.51 × 10^–9^, enrichment number 27) (See Figure 2). Among the three marked pathways, dre03010 Ribosome has the highest degree of enrichment. Note that the 27 differential genes in the ribosomal pathway are all upregulated. Their expression levels were upregulated by at least two times and the most upregulated by 2.8 times (see Figure 3).

### 3.5. Analysis of the Protein Interaction Network

**Figure 4:**
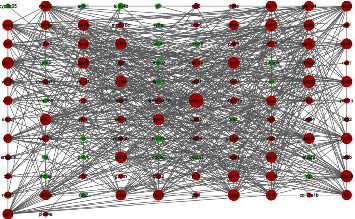
Protein-protein interaction network of differentially expressed genes. Each node represents the protein corresponding to the differentially expressed gene (green means downregulation, while red means upregulation). The edges represent the interactions between the proteins.

**Figure 5:**
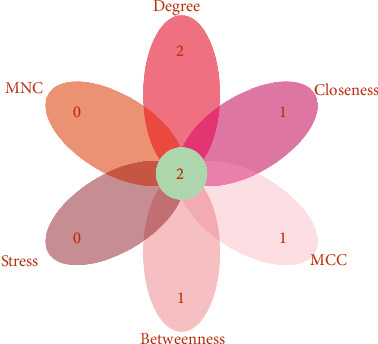
Venn diagram of hub genes obtained using different algorithms. The number marked in the center represents the number of hub genes obtained by the intersection of the six algorithms.

Based on the STRING online database and Cytoscape software, we have drawn a protein-protein interaction network diagram that includes 112 differential genes (24 downregulated and 88 up-regulated) and 608 protein-protein interaction networks, as shown in Figure 4. The larger the node, the more interactive the relationship with other nodes. As shown in Figure 4, mrto4, rpl23a, uba52, and rpl3 are the most prominent nodes. To identify potential hub genes in the network, the Cytoscape plug-in, CytoHubba, used six algorithms to calculate and screen the protein–protein interaction network and select the top 10 genes of each algorithm (Table 4). The results of all topological measurements have a relatively high degree of overlap. We used the Venn diagram to intersect the results obtained by the six algorithms and finally screen out uba52 and rpl23a, as shown in Figure 5. These two genes have a more robust interaction relationship than other genes in the entire protein–protein interaction network. They may play a more critical role in the reproduction process than other genes.

## 4. Discussion

P. canaliculata was introduced into Guangdong for breeding in the early 1980s [9]. Then, in the early 1990s, they were introduced across multiple provinces and cities, with the distribution area gradually extending to Hunan and other places. The reproduction of apple snails is affected by temperature [10, 11]. The suitable temperature for breeding and hatching is 22-32°C, and the eggs usually cannot hatch after the temperature is lower than 15°C. Since the annual average temperature in Guangdong Province is about 19-24°C, and the average temperature in January is about 16-19°C, apple snails can reproduce in Guangdong three generations a year [8]. Meanwhile, since the annual average temperature in Hunan Province is 16-18.5°C, and the average temperature in January is about 4-7°C, apple snails cannot reproduce for three generations a year in Hunan because of low temperature. Apple snails consume energy to adapt to a low-temperature environment, so their energy for reproduction is reduced, which means their fecundity is lower than that of apple snails in Guangdong.

The reproductive characteristics of apple snails show huge differences between populations, partly due to phenotypic plasticity and partly due to genetic variation [12, 13]. The reproduction of snails is a complex process involving many genes. In this study, 1,546 genes were identified by analyzing G_O and H_O. The results of the KEGG enrichment analysis based on differential genes and protein interaction network analysis showed that the ribosomal pathways of *rpl23a* and *uba52* and two ribosomal-related pivot genes are essential pathways and key genes that affect the reproductive ability of apple snails.

Ribosomes are important sites for protein synthesis. The ribosomes of eukaryotes are composed of 60S large subunits and 40S small subunits. A 60S large subunit consists of three rRNA molecules (25SrRNA, 5.8SrRNA, and 5SrRNA) and 46 proteins, while the 40S small subunit includes one rRNA (18SrRNA) and 33 proteins [14–16]. Ribosomal Protein (RP) is a collective term for all proteins forming ribosomes. It is widely distributed in various tissues and forms ribosomes together with ribonucleic acid. It plays a vital role in protein biosynthesis. Many ribosomal proteins form ribosomes, participate in protein biosynthesis, and have functions independent of protein biosynthesis. Studies have shown that ribosomes are closely related to cell growth, differentiation, and embryonic development [17, 18]. In this experimental study, the differential genes involved in the ribosomal signaling pathway in the H_O were all downregulated, 23 of which were ribosomal protein genes. The downregulation of ribosomal protein genes will reduce the production of corresponding ribosomal proteins, thereby reducing the number of ribosomes in the cell. This fact indicates that the ovaries of apple snails in Hunan Province, due to the downregulation of differential gene expression, affect the protein biosynthesis of ovarian tissues, thereby affecting the normal physiological functions of the ovaries and showing poor reproductive ability.

The *rpl23a* (ribosomal protein l23a) gene encodes a ribosomal protein, which is a component of the 60S subunit. As an indispensable part of eukaryotic ribosomes, the *rpl23a* gene can improve the catalytic ability of rRNA to synthesize proteins [19], and it plays a vital role in protein synthesis, folding, and arrangement [20]. *Uba52* (ubiquitin a-52 residue ribosomal protein fusion product 1) gene is an essential member of the ubiquitin family. Ubiquitin is a small protein with 76 amino acids and about 8.6 kDa. It is ubiquitous and highly conserved in eukaryotes. Ubiquitin can play a crucial role in tissue remodeling and development, gametogenesis and maturation, fertilization, and early pregnancy through the ubiquitin-proteasome pathway (UPP) [21]. Kobayashi's research on mouse embryos confirmed that *uba52* regulates ribosomal protein complexes and simultaneously provides *rpl40* and ubiquitin to ribosomes. *Uba52-*deficient mice die during embryogenesis, indicating that *uba52* can maintain embryos' developmental function [22]. Mao et al. found that the *uba52* gene is essential for early embryogenesis in pigs [23].

By comparing the transcriptome and bioinformatics analyses of G_O and H_O, we predict that the ribosome signaling pathway may be closely related to snail reproduction. In addition, we determined that *rpl23a* and *uba52* genes play an essential role in snail reproduction. Of course, the specific mechanisms and functions of these pathways and genes need to be verified through many biological experiments to provide solid evidence for understanding the reproductive mechanism of Pomacea canaliculata.

## Figures and Tables

**Table 1:** Absolute fecundity of Pomacea canaliculata in Guangdong and Hunan.

Area	Absolute fecundity (eggs/egg mass)	Range
Guangdong	282.34 ± 125.92^a^	69-799
Hunan	163.52 ± 80.87^b^	54-340

**Table 2:** Relative fecundity of Pomacea canaliculata in Guangdong and Hunan.

Area	Weight (g)	Egg masses	Eggs	Relative weight fecundity (eggs/weight)
Guangdong	23.92 ± 2.22^a^	3.67 ± 1.15^a^	653.33 ± 60.12^a^	27.59 ± 4.67^a^
Hunan	27.51 ± 18.36^a^	1.67 ± 0.58^a^	246.33 ± 20.55^b^	11.80 ± 6.80^b^

**Table 3:** Comparison results of clean reads and reference genomes.

Sample number	Total reads	Clean bases/G	Mapped ratio/%	Uniq mapped reads	Uniq mapped ratio/%
G_O1	55420990	8.31	85.91	46567375	84.02
G_O2	48191858	7.23	85.34	40141606	83.3
G_O3	49830396	7.47	84.77	41284342	82.85
H_O1	54695332	8.2	81.06	43277495	79.12
H_O2	53698116	8.05	80.51	41838227	77.91
H_O3	50923560	7.64	83.9	41248544	81.0

**Table 4:** Top 10 hub genes obtained by different algorithms.

Gene	Degree	Gene	Closeness	Gene	MCC	Gene	Betweenness	Gene	Stress	Gene	MNC
mrto4	39	mrto4	56.83333	rpl23a	9.223e13	mrto4	1351.4171	mrto4	11736	mrto4	39
rpl23a	32	rpl23a	51.25	rps11	9.223e13	nhp2l1a	867.04406	nsa2	4822	rpl23a	31
uba52	30	uba52	50.83333	rpl15	9.223e13	uba52	735.93096	fbl	3334.0	rpl3	30
rpl3	30	rpl3	50.25	rpl18	9.223e13	nsa2	480.52399	rpl23a	3232.0	rps11	29
rps11	29	rps11	49.91667	rpl3	9.223e13	dkc1	388.92945	nhp2l1a	2786.0	rpl15	28
rpl15	28	nsa2	49.75	rpl4	9.223e13	fbl	365.27046	uba52	2644.0	rps2	28
rpl4	28	rpl15	49.41667	rpl10a	9.223e13	nop56	331.98893	nop56	2312.0	uba52	28
rps2	28	rpl4	48.75	rps2	9.223e13	rpl23a	327.47269	pes	2286.0	rpl4	28
rpsa	27	rpl2	48.75	uba52	9.223e13	gnb2l1	302.95149	dkc1	2178.0	rpl10a	27
rps7	27	rpl10a	48.25	rpl31	9.223e13	pes	164.6678	rpl3	2074.0	rpl18	27

## Data Availability

The data of this study are available from the corresponding author.
